# miR-19b regulates *hTERT* mRNA expression through targeting *PITX1* mRNA in melanoma cells

**DOI:** 10.1038/srep08201

**Published:** 2015-02-03

**Authors:** Takahito Ohira, Sunamura Naohiro, Yuji Nakayama, Mitsuhiko Osaki, Futoshi Okada, Mitsuo Oshimura, Hiroyuki Kugoh

**Affiliations:** 1 Department of Biomedical Science, Institute of Regenerative Medicine and Biofunction, Graduate School of Medical Science, Tottori University, Yonago, Tottori 683-8503, Japan; 2 Division of Functional Genomics, Research Center for Bioscience and Technology, Tottori University, Yonago, Tottori 683-8503, Japan; 3 Division of Pathological Biochemistry, School of Life Science, Faculty of Medicine, Tottori University, Yonago, Tottori 683-8503, Japan; 4 Chromosome Engineering Research Center, Tottori University, Yonago, Tottori 683-8503, Japan

## Abstract

Human telomerase reverse transcriptase (*hTERT*) plays a crucial role in cancer development. We previously identified paired-like homeodomain1 (*PITX1*) as an *hTERT* suppressor gene. However, the underlying mechanisms that are involved in the regulation of *PITX1* remain unknown. Here, we report that the microRNA-19b (miR-19b) regulates *hTERT* expression and cell proliferation through inhibition of *PITX1*. Compared with normal melanocyte cells, miR-19b expression was higher in most melanoma cells and was accompanied by downregulation of *PITX1*. Moreover, overexpression of miR-19b inhibited *PITX1* mRNA translation through a miR-19b binding site within the 3′UTR of the *PITX1* mRNA. Our combined findings indicate the participation of miR-19b as a novel upstream effector of *hTERT* transcription via direct targeting of *PITX1*.

Human telomerase reverse transcriptase (*hTERT*), which is specifically activated in most cancer cells and germ cells, plays an essential role in the immortality of cancer cells via regulation of telomere length by telomerase enzyme activity^1^. Furthermore, *hTERT* has noncanonical functions in addition to that of maintaining telomere length. It was recently reported that hTERT acts as a transcriptional modulator of Wnt/beta-catenin and nuclear factor-κB (NF-κB) signaling pathways, resulting in the enhanced expression of Wnt and NF-κB target genes that facilitate cancer promoting functions such as proliferation and resistance to apoptosis^2^,^3^. However, the signaling mechanism that controls *hTERT* transcriptional regulatory factors remains unclear.

We previously identified paired-like homeodomain1 (*PITX1*) as a novel *hTERT* suppressor gene. PITX1 represses *hTERT* transcription through direct binding to its promoter, and eventually leads to the inhibition of telomerase activity and of cell proliferation^4^. In addition, *PITX1* also acts as a negative regulator of the RAS pathway through RAS protein activator like 1 (*RASAL1*) gene, a member of the RAS GTPase-activating protein family^5^. Downregulation of PITX1 was reported in various types of human cancer, including colon, prostate, bladder, lung, and gastric cancers, Barrett's-adenocarcinoma, oral tumors and malignant melanoma^5^,^6^,^7^,^8^,^9^,^10^. This evidence suggests that *PITX1* plays a crucial role in cancer development. However, as yet little is known regarding PITX1 upstream regulatory mechanisms.

MicroRNAs (miRNAs) are small non-coding RNA molecules that regulate gene expression through induction of direct degradation of mRNA or through translational repression by binding to the 3′ untranslated region (3′ UTR) of the mRNA of target genes. miRNAs are involved in fundamental cellular functions such as apoptosis, development, differentiation, proliferation and carcinogenesis. In cancer, miRNAs function as gene regulatory molecules, acting as oncogenes or tumor suppressors. Aberrant overexpression of oncogenic miRNAs downregulates tumor suppressor genes or other genes involved in cell differentiation, thereby contributing to tumor development by stimulating proliferation, immortalization and invasion^11^. Indeed, some microRNAs have been reported to be directly involved in and be a key factor for cancer development, and these miRNAs may be ideal biomarkers of and therapeutic targets for cancer^12^,^13^. For example, oncogenic miR-135b was reported to promote tumor transformation and progression in colon cancer^14^. miRNAs have also been implicated in the regulation of *hTERT* expression. miR-21 regulates *hTERT* expression via the phosphoinositide 3-kinase (*PI3K*) signaling pathway by directly targeting the mRNA of the *PI3K* signaling inhibitor gene, phosphatase and tensin homolog deleted from chromosome 10 (*PTEN*) in hypertrophic scar fibroblasts (HSFBs)^15^. However, the involvement of miRNAs in the *hTERT* regulatory network that interconnects with oncogenesis pathways is not well understood.

miR-19b is included in both the miR-17-92 and miR-106-363 clusters. These miRNA clusters carry out pleiotropic functions during both normal development and malignant transformation, as they act to promote proliferation and sustain cell survival^16^,^17^. miR-19b was identified as the key oncogenic component of a miRNA cluster in a B-cell transformation model^18^. miR-19b also coordinates a PTEN/PI3K pathway that influences cell survival in mouse leukemia and inhibits mRNA translation of the tumor suppressor *PTEN* gene in human breast cancer^19^,^20^.

In the present study, we show that *PITX1* mRNA is a direct target of miR-19b and that downregulation of *PITX1* by miR-19b ultimately induces enhancement of *hTERT* mRNA expression. Moreover, overexpression of miR-19b and a decrease in PITX1 at both the mRNA and protein levels were observed in many malignant melanoma cell lines and patient samples compared to normal melanocytes. These findings provide evidence that suggests that miR-19b might regulate cancer development through telomerase-dependent pathways.

## Results

### *PITX1* mRNA is a target of miR-19b

**Figure 1:**
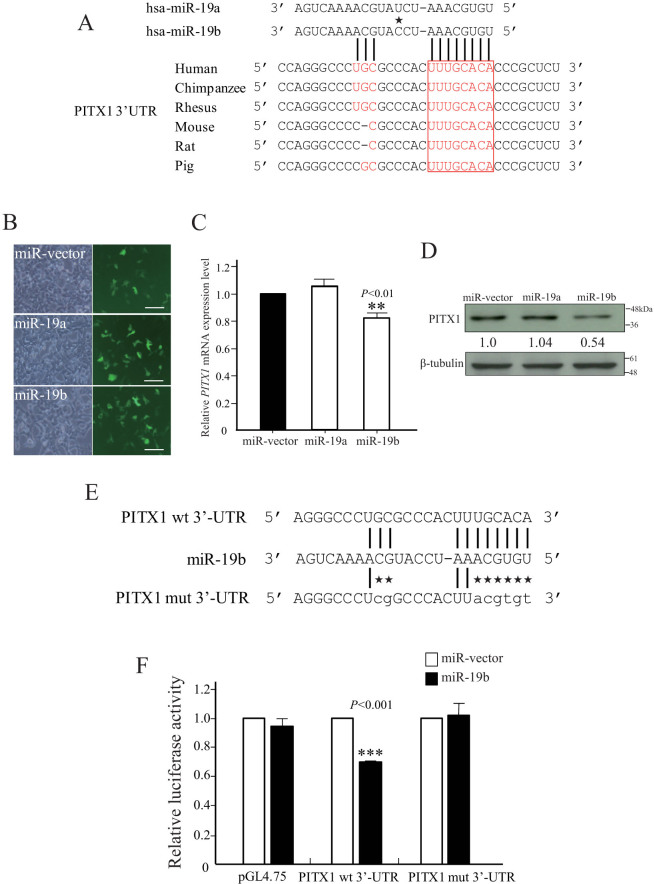
*PITX1* is a target of miR-19b. (A) Sequence alignment of the miR-19a/b nucleotide sites with the 3′UTR of *PITX1* mRNA of different species. The sequences of miR-19a/b that are complementary to *PITX1* 3′-UTR mRNA sequences are shown in red. Sequences complementary to the eight seed nucleotides at the 5′ end of miR-19a/b are boxed. A single different nucleotide is indicated by a star. (B) 293T cell lines were transiently transfected with pCMV-miR control and miR-19a/b expression plasmid vectors. Transfection efficiency was monitored after 48 h by fluorescence analysis of vector encoded GFP (right panels). Phase contrast images are shown at left. Scale bars: 100 µm. (C) qRT-PCR analysis of relative *PITX1* mRNA expression levels in miR-19a/b and miR-vector control transiently transfected 293T cells. Expression in the vector control cells was arbitrarily set at 1. *GAPDH* mRNA expression was used as the internal control. Data are presented as means ± S.D. of three independent experiments (**P<0.01). (D) Western blotting analysis of the protein level of PITX1 in 293T cells at 48 h after transient transfection with miR-19a/b or control miR-vector. The expression levels of PITX1 were normalized to the levels of β-tubulin. Cropped blots were used in this figure. Original full-length blots are presented in Supplementary Fig S5. (E) Alignment of the miR-19b nucleotide sequence with the wild type and mutated target sites of the *PITX1* mRNA 3′-UTR region (PITX1 wt 3′-UTR and PITX1 mut 3′-UTR respectively) that were used to construct luciferase reporter plasmids. Mutated nucleotides are indicated by stars. (F) 293T cells were transfected with miR-19b or the miR-control vector and were co-transfected with the control luciferase reporter plasmid pGL4.75 or with this reporter plasmid containing the PITX1 wt 3′-UTR or the PITX1 mut 3′-UTR. Luciferase activity was assayed 48 h later. Renilla luciferase values were normalized to total protein concentration. Luciferase activity in each miR-vector-transfected cell was arbitrarily set at 1. Data are presented as means ± S.D. of three independent experiments (***P<0.001).

We previously identified *PITX1* as a novel *hTERT* suppressor gene. We therefore further investigated the regulation of *PITX1* in order to understand the molecular mechanism of telomerase-dependent pathways in cancer development. To determine if *PITX1* mRNA was targeted by miRNA, we screened for a candidate miRNA that might bind to the 3′ UTR of the *PITX1* mRNA using the TargetScan Human 6.2 software. We identified miR-19a and miR-19b as a miRNA that includes a seed sequence at the 5′ end that is complementary to a sequence within the 3′UTR region of *PITX1* mRNA (nucleotides 912-919). Moreover, the *PITX1* mRNA regions complementary to these 8 nt seed sequences of miR-19a/b are highly conserved among different species (Fig 1A). To determine whether miR-19a/b regulates the translation of *PITX1* mRNA, we first generated 293T cells in which a miR-19a/b expressing or control vector (miR-vector) was transiently overexpressed. These vectors also express GFP, which was used to monitor transfection efficiency. As shown Fig 1B, fluorescence microscopic analysis after 24 h indicated a high transfection efficiency for both the miR-19a/b and the miR-vector. 293T cells were chosen for these experiments because quantitative reverse transcription PCR (qRT-PCR) analysis indicated that endogenous miR-19a/b was expressed at a low level in 293T cells compared to normal human epidermal melanocytes (NHEMs) (supplementary Fig S1 online). Transient overexpression of miR-19b induced a significant decrease in *PITX1* mRNA levels compared to miR-19a- or miR-vector-transfected cells (Fig 1C, *P* < 0.01). Furthermore, Western blotting analysis of these cells at 48 h after transfection showed that the protein level of PITX1 was markedly reduced in miR-19b overexpressing cells compared with the cells transfected with miR-19a or control vector without miR-19b (Fig 1 D). miR-19a and miR-19b differ only a single nucleotide at position 11 from 5′ end (Fig 1A). This sequence position may play a crucial role of target recognition^16^. Therefore, we focused here on miR-19b.

To further validate the putative site of miR-19b initial binding within the 3′UTR of *PITX1* mRNA, we generated full length of PITX1-3′UTR luciferase reporter vectors that contained the predicted, wild type (wt) miR-19b binding site (PITX1 wt 3′-UTR) or a mutated version (PITX1 mut 3′-UTR) (Fig 1E). These vectors, or the control pGL4.75 vector, were individually co-transfected with either the miR-19b or the miR control vector and luciferase activity was assayed 48 h later. Overexpression of the miR-19b, but not of the control miR-vector, decreased the luciferase activity of the PITX1 wt 3′-UTR vector-transfected cells but not that of the PITX1 mut 3′-UTR vector- or of the control pGL4.75 vector-transfected cells (*P*<0.001, Fig 1F). These results provide evidence that miR-19b directly repressed *PITX1* translation through a specific 3′UTR mRNA binding sequence.

### Downregulation of PITX1 expression by miR-19b leads to activation of *hTERT* transcription in 293T cells

**Figure 2:**
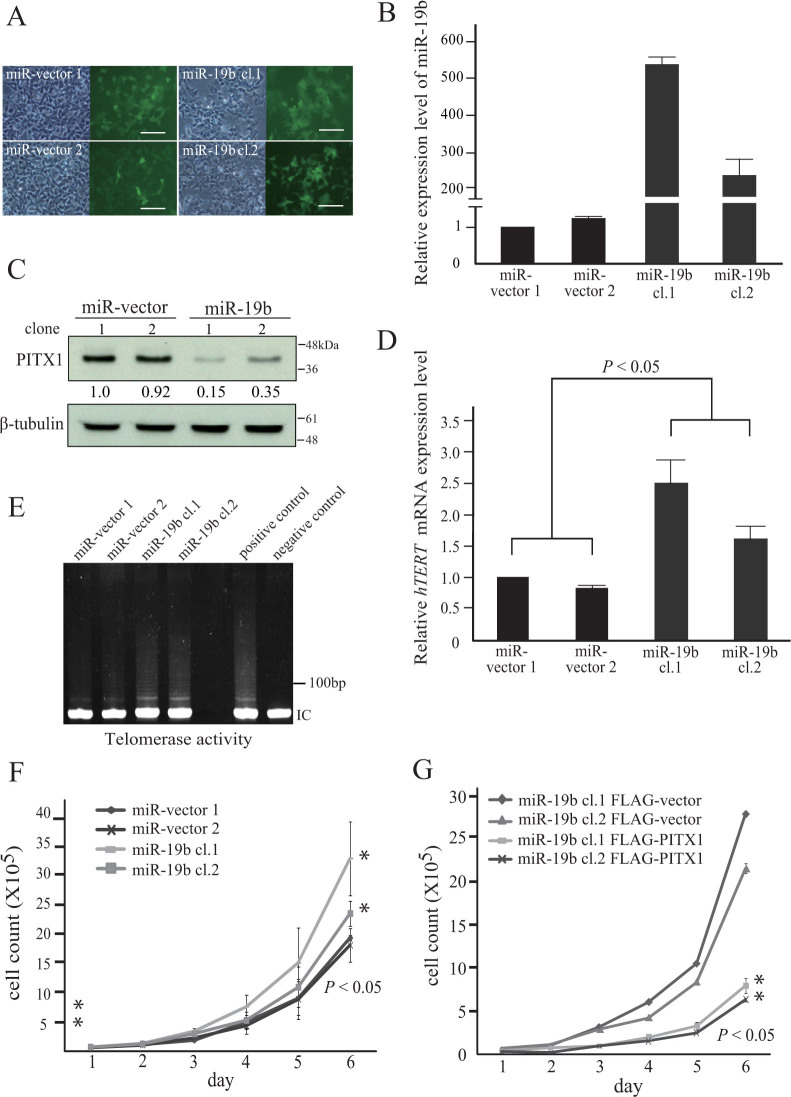
miR-19b regulates *hTERT* transcription activity through direct targeting of *PITX1* mRNA. (A) Established 293T cell lines (cl.1 and cl.2) that stably express miR-19b or the control miR-vector were microscopically analyzed. Phase contrast images are shown at left. The GFP transgene that was stably expressed in these cells was analyzed by fluorescence microscopy (right panels). Scale bars: 100 µm. (B) qRT-PCR analysis of relative miR-19b expression levels in miR-19b stably expressing 293T cell lines and control cells. Data were normalized to *U6* control. The bars correspond to means ± S.D. (C) Western blotting of the PITX1 protein level in cloned cell lines stably expressing miR-19b or the miR-vector control. The expression levels of PITX1 were normalized to the levels of β-tubulin. Cropped blots were used in this figure. Original full-length blots are presented in Supplementary Fig S6. (D) qRT-PCR analysis of *hTERT* mRNA expression levels in miR-19b stably transfected cell lines relative to vector control cells. Data were normalized to *GAPDH* mRNA control. The expression level in miR-vector 1 cells was arbitrarily assigned as 1. The bars correspond to means ± S.D. of three independent experiments (*P<0.05). (E) Telomerase activity was measured using TeloChaser kit. IC: internal control for PCR and loading. Telomerase activity of 2.5 × 10^4^ HeLa cells extract served as a positive control. Negative control was heat inactivated HeLa cell extract. Cropped blots were used in this figure. Original full-length gel images are presented in Supplementary Fig S9. (F) Cell number of miR-19b stably transfected cell lines and vector controls over 6 days. The bars correspond to means ± S.D. of three independent experiments (*P<0.05). (G) Cell number of miR-19b stably transfected cell lines that were co-transfected with the control FLAG-vector or with exogenous FLAG-PITX1, over 6 days. The bars correspond to means ± S.D. of three independent experiments (*P<0.05).

To further explore the effects of miR-19b on *PITX1* transcription, we generated two 293T cell lines that each stably overexpressed miR-19b (cl.1 and cl.2) or the control miR-vector (miR-vector 1 and 2) (Fig 2A). Significantly increased expression of miR-19b in the miR-19b transfected cells compared to control transfected cells was confirmed using qRT-PCR (Fig 2B). Western blot analysis indicated that the protein level of PITX1 was markedly reduced in the miR-19b overexpressing clones compared with that in the miR-vector control clones (Fig 2C). Since we previously demonstrated that PITX1 can suppress *hTERT* expression in a transcription-dependent manner^4^, we next determined whether miR-19b can lead to the regulation of *hTERT* transcription through an effect on the downregulation of PITX1. We therefore investigated *hTERT* transcription in clones that overexpress miR-19b and found that *hTERT* transcription levels were significantly increased in clones that overexpressed miR-19b compared with their controls (*p*<0.05, Fig 2D). The expression of *hTERT* mRNA largely parallels telomerase activity. To investigate whether telomerase activity was also increased by overexpression of miR-19b, we measured using a telomerase activity detection kit (TeloChaser) based on the Stretch PCR method. As observed in Fig 2E and Supplementary Fig S2, overexpressing clones of miR-19b resulted in a 1.5- to 1.7-fold increase in telomerase activity. However, these cells did not show the elongation of telomere length. Since it is known that the telomerase activity level is not always associated with telomere length. Telomerase activity was detected in parental 293T and A2058 cells in this study. Thus, the change of telomerase activity mediated by miR-19b may not always affect the telomere length.

Furthermore, an increase in the growth rate of the miR-19b overexpressing clones compared to the control clones was observed (*P*<0.05, Fig 2F). To confirm the above described effects of miR-19b, FLAG-tagged PITX1 expression plasmids that lack the PITX1 3′UTR region, or the control FLAG-vector, were transfected into miR-19b stably expressing clones (miR-19b FLAG-PITX1 and miR-19b FLAG-vector respectively). Endogenous PITX1 in miR-19b FLAG-vector cells and endogenous PITX1 and exogenous FLAG-PITX1 expression levels in miR-19b FLAG-PITX1 cells were analyzed by Western blotting (see Supplementary Fig S3A online). In addition, downregulation of *hTERT* expression in 293T overexpressing cells of miR-19b by introduction of FLAG-PITX1 decreased telomerase activity by 57–77% (see Supplementary Fig S3 online). Additionally, introduction of FLAG-PITX1 into miR-19b cells, in which endogenous PITX1 was decreased by miR-19b, inhibited cell growth compared with that in miR-19b FLAG-transfected cells (Fig 2G). These combined results suggest that the functional effects of miR-19b regulation of PITX1 levels are activation of *hTERT* transcription and enhancement of cell proliferation.

### High miR-19b expression is correlated with downregulation of PITX1 in melanoma cell lines and tissue samples

**Figure 3:**
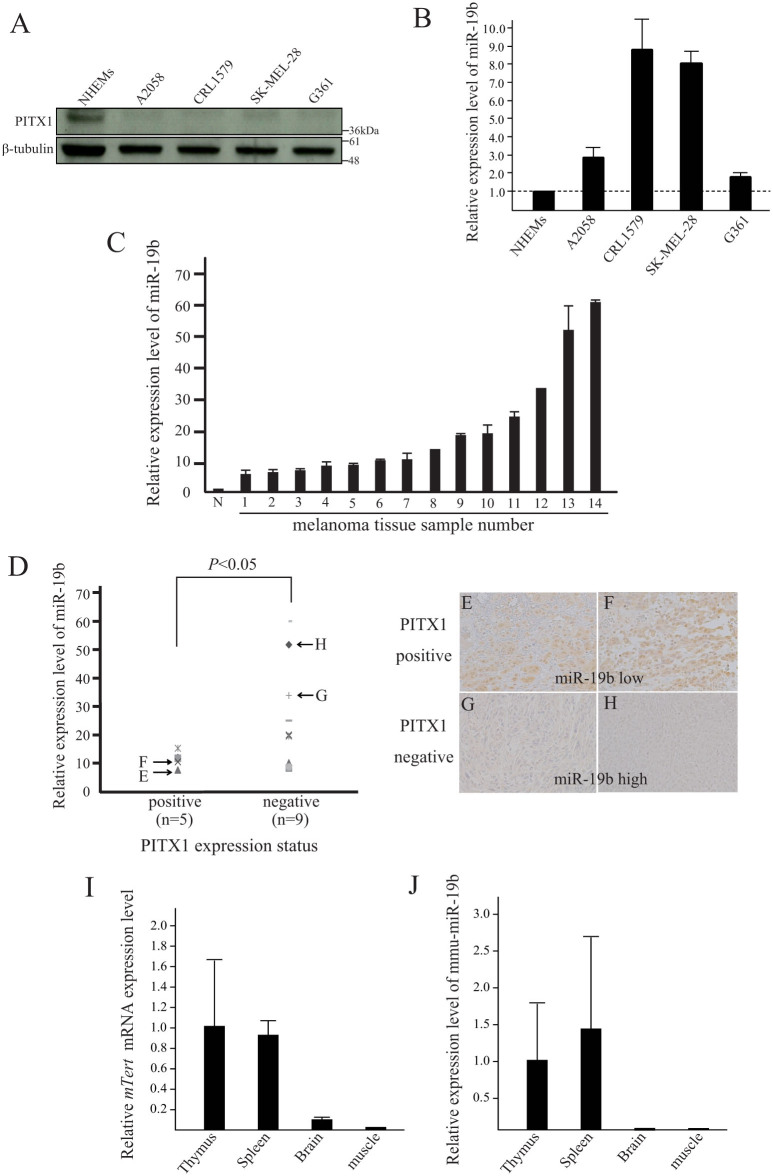
miR-19b and PITX1 expression in human melanoma cells and primary tissues. (A) Western blotting analysis of PITX1 protein expression in melanoma cell lines and normal human epidermal melanocytes (NHEMs). β-tubulin was used as a protein loading control. Cropped blots were used in this figure. Original full-length blots are presented in Supplementary Fig S7. (B) qRT-PCR analysis of relative miR-19b expression levels in human melanoma cell lines and NHEMs. Data were normalized to U6 control. Results are expressed relative to the value of NHEMs that were assigned a value of 1. The bars correspond to means ± S.D. (C) qRT-PCR analysis of miR-19b expression levels in human melanoma clinical tissue samples relative to that in NHEMs (N), which was assigned a value of 1. Data were normalized to U6 control. The bars correspond to means ± S.D. (D) Relationship between miR-19b expression and PITX1 positive or negative expression in melanoma clinical samples (*P<0.05). miR-19b expression level was detected by qRT-PCR (Fig. 3C) and PITX1 protein status was determined by immunohistochemical staining (IHC). (E-H) Representative positive and negative immunohistochemical staining of PITX1 in low-expressing miR-19b melanoma tissues (E, F) and high-expressing miR-19b melanoma tissues (G, H). (I-J) qRT-PCR analysis of mmu-miR-19b expression levels in mouse *mTert*-positive (thymus and spleen) and –negative (brain and muscle) tissues. Data were normalized to *mouse Gapdh* control. The bars correspond to means ± S.D.

We next performed qRT-PCR analysis to determine the expression level of miR-19b in melanoma cell lines in which PITX1 protein expression is known to be lower than that of normal cells. Consistent with a previous report^10^ PITX1 protein expression in the human melanoma cell lines A2058, CRL1579, SK-MEL-28 and G361 was lower than that in NHEMs as assayed by Western blotting (Fig 3A). miR-19b expression was much higher in all melanoma cell lines compared to its expression level in NHEMs (Fig 3B). Furthermore, qRT-PCR analysis indicated higher expression of miR-19b in all fourteen human clinical melanoma specimens than that in NHEMs (Fig 3C). These results suggested that the higher endogenous level of miR-19b in human melanoma cells may be responsible for the downregulation of PITX1 protein expression in these cells.

To further determine the relationship between the expression level of miR-19b and that of PITX1 *in vivo*, we immunohistochemically analyzed PITX1 protein expression status in each of the fourteen clinical melanoma tissue specimens in which miR-19b expression levels had been assayed (Fig. 3C). Five of the melanoma cases were PITX1-positive (melanoma tissue sample number 1, 5, 6, 7, 8) and nine cases were PITX1-negative (melanoma tissue sample number 2, 3, 4, 9, 10, 11, 12, 13, 14) (Fig. 3C and D). Representative cases are shown in Figure 3E-H (melanoma tissue sample number 1, 5, 12, 13). When PITX1 expression status was plotted against miR-19b expression level, high miR-19b expression tended to be associated with decreased PITX1 expression (*P*<0.05) (Fig. 3D). These data provide strong evidence of a functional link between miR-19b and PITX1 expression levels in melanoma tissues.

To clarify whether miR-19b expression correlated with telomerase activity in mice tissues expressing mouse *Tert* (*mTert*), we performed expression analysis of mouse miR-19b (mmu-miR-19b) in mTert-positive (thymus and spleen) and –negative (brain and muscle) tissues^21^. mmu-miR-19b was detected in thymus and spleen, but not in brain and muscle tissues (Fig 3I and J). Thus, this result provides evidence that miR-19b may play a significant role in the regulation of telomerase activity.

### Knockdown of miR-19b leads to increased expression of PITX1

**Figure 4:**
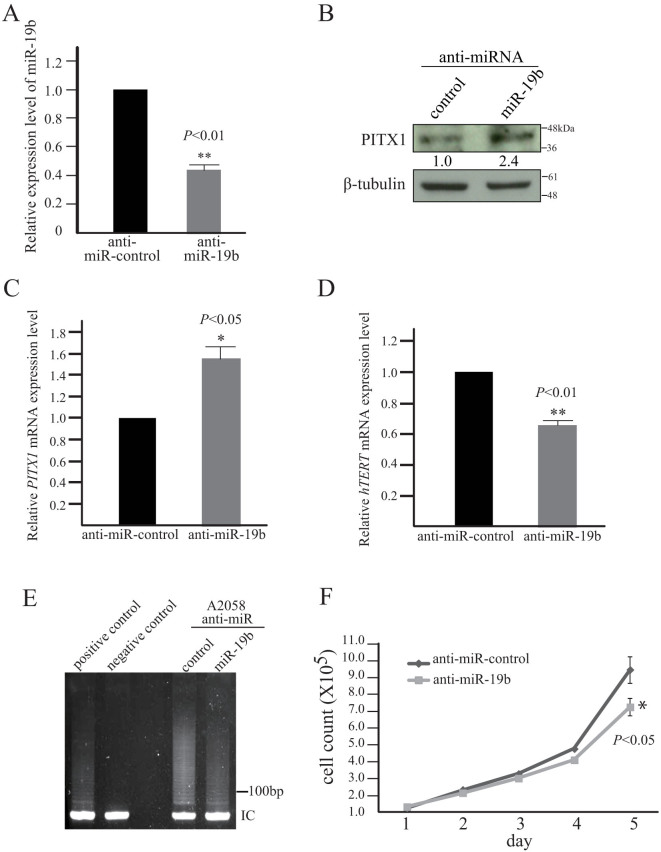
Knockdown of miR-19b results in suppression of *hTERT* transcription and inhibition of cell growth through upregulation of PITX1 expression. (A) qRT-PCR analysis of relative miR-19b expression levels in anti-miR control or anti-miR-19b oligonucleotide transfected A2058 cells. Data were normalized to U6 control. The level in the control cells was assigned a value of 1. The bars correspond to means ± S.D. of three independent experiments (**P<0.01). (B) The protein expression level of PITX1 measured by western blotting at 48 h after transfection of A2058 melanoma cells with anti-miR control or anti-miR-19b oligonucleotides. The expression levels of PITX1 were normalized to the levels of β-tubulin. Cropped blots were used in this figure. Original full-length blots are presented in Supplementary Fig S8. (C and D) qRT-PCR analysis of relative *PITX1* mRNA expression levels (C) and relative *hTERT* mRNA expression levels (D) in A2058 melanoma cells transfected with anti-miR control or anti-miR-19b oligonucleotides. Data were normalized to *GAPDH* mRNA levels. The level in the control cells was assigned a value of 1. The bars correspond to means ± S.D. of three independent experiments (*PITX1* mRNA, *P<0.05; *hTERT* mRNA **P<0.01). (E) Telomerase activity was measured using TeloChaser kit. IC: internal control for PCR and loading. Telomerase activity of 2.5 × 10^4^ HeLa cells extract served as a positive control. Negative control was heat inactivated HeLa cell extract. Cropped blots were used in this figure. Original full-length gel images are presented in Supplementary Fig S10. (F) Cell number of anti-miR control or anti-miR-19b oligonucleotide-transfected A2058 cells over 5 days. The bars correspond to means ± S.D. of three independent experiments (*P<0.05).

To further examine the suppressive effects of miR-19b on *PITX1* under more physiological conditions, we knocked down endogenous miR-19b expression in human melanoma A2058 cells using anti-miR-19b oligonucleotides. Knockdown of miR-19b in A2058 cells reduced its expression to 44% of that of control cells (*P*<0.01, Fig 4A), and resulted in a 2.4 and 1.6-fold increase in PITX1 protein and mRNA expression, respectively, compared to that of control (Fig 4B, C). In contrast, knockdown of miR-19b resulted in significantly reduced *hTERT* mRNA expression (reduced to 67% of control) (*P*<0.01, Fig 4D). Furthermore, the knockdown of miR-19b transcription resulted in a reduction of telomerase activity by 51% (Fig 4E and Supplementary Fig S4 online), and miR-19b knockdown also led to a decrease in A2058 cell proliferation compared to control cells (P<0.05) (Fig 4F). These data demonstrate that miR-19b decreases *PITX1* expression, which modulates telomerase dependent pathways.

## Discussion

hTERT is the principal component for the control of telomerase activity that is crucial for cellular immortalization and cancer progression. *hTERT* transcription is controlled by positive and negative transcription factors^22^,^23^. We previously identified *PITX1* as an *hTERT* suppressor gene^4^. However, the precise molecular mechanism that underlies the *hTERT* transcriptional network remains unclear. In this study, we found that miR-19b directly targets *PITX1* mRNA and leads to an increase in *hTERT* mRNA levels. A previous study showed that miR-21 stimulated *hTERT* transcription through direct targeting of *PTEN*^15^, which is a tumor suppressor gene that inhibits the PI3K pathway^24^. However, the molecular mechanism by which *hTERT* is regulated by the PI3K pathway remains poorly understood^25^. In contrast, we demonstrated that PITX1 directly suppresses transcription of *hTERT* by binding to its promoter region^4^. To our knowledge, the present study is the first report to provide evidence that *hTERT* transcription is modulated through interaction between a microRNA (miR-19b) and a suppressor gene (*PITX1*). Thus, our results suggest that oncogenic miR-19b functions may play an important role in one of the telomerase pathways that regulate cancer progression. However, regulation of telomerase activity in normal cells may not be simply explained by the miR-19b-PITX1 pathway, and possibly involve in multiple factors. It has been reported that no remarkable change in the mean telomere length was found in human foreskin fibroblasts with ectopic hTERT expression^26^. Authors have concluded in the study that the result showing no change in the average telomere length are in agreement with the maintenance of the shorter telomeres preventing chromosome instability and cell senescence.

Human miR-19b is included in the miR-17-92 and the miR-106-363 clusters. These double clusters are located at the chromosomal region 13q31.3 or Xq26.2, at which coding miRNAs are frequently overexpressed in malignant cancers, including diffuse B-cell lymphomas, follicular lymphomas, Burkitt's lymphomas, lung carcinoma and human T-cell leukemia^27^,^28^,^29^,^30^,^31^. Our findings are consistent with these data as they provide evidence that miR-19b is crucial for the phenotypes of transformed cells and is a key oncogenic factor in the multistep processes of neoplastic development.

It was reported that miR-19b promotes *PI3K* pathway signaling through inhibition of PTEN expression^18^,^19^. The *PI3K* intracellular signaling pathway is involved in the regulation of anti-apoptosis, cell proliferation and cell growth^25^. Our results showed that knockdown of miR-19b leads to inhibition of proliferation of the melanoma cell line A2058 (Fig 4E). However, A2058 cells lack the *PTEN* gene^32^. These findings suggest that the inhibition of cell growth resulting from knockdown of miR-19b in melanoma cells is independent of *PI3K* signaling pathway regulation by *PTEN*. Therefore, miR-19b mediated *hTERT* activation through the targeting of PITX1 may involve another oncogenesis pathway such as NF-κB or the Wnt signaling pathway, which can be activated by hTERT expression in melanoma cells.

Overexpression of the miR-17~92 cluster, which contain miR-19b, is frequently observed in human B-cell lymphoma, and cooperate with the c-Myc oncogene in a mouse model of B-cell lymphoma^16^,^33^,^34^. In addition, miR-19b is also overexpressed in human lung cancer and promotes proliferation of lung epithelial progenitor cells, eventually induced abnormal lung phenotype in the transgenic mice^35^,^36^. Moreover, it has been reported that miR-19b potentiates NF-κB activity in human and mouse cells^37^. Thus, these results suggested that miR-19b could function similar between human and mouse.

Mutations of the serine/threonine-protein kinase BRAF have been observed in 50% of malignant melanomas, and result in activation of mitogen activating protein kinase (MAPK) pathways. Therefore, this pathway is an important target for drug discovery and development^38^. The expression of miR-19b was shown to be increased by the introduction of mutated BRAF into normal thyroid cells^39^. Our present data showed that the expression level of miR-19b was increased in the melanoma cell lines A2058, CRL1579, SK-MEL-28 and G361 (Fig 4A, B), which have been shown to express BRAF-mutants^32^,^40^. These results suggest that BRAF mutation may trigger an increase in the levels of miR-19b expression. Indeed, miR-19b is overexpressed in thyroid, colorectal and lung cancer that express mutant BRAF^39^,^41^,^42^,^43^,^44^,^45^,^46^,^47^. In addition, *PITX1* expression is reduced in these various types of human cancers^5^,^6^,^7^. It is therefore likely that reduction of PITX1 and overexpression of miR-19b not only play a role in the development of malignant melanoma, but may also play a role in various other types of cancers. Further study involving a detailed analysis of the regulation of miR-19b transcription may contribute greatly to novel anti-cancer drug discovery and therapy.

In conclusion, our study combined with previous studies showed that miR-19b controls at least two distinct oncogenic signaling (PI3K and BRAF/MAPK) and telomerase dependent pathways that are involved in cancer progression. Further studies aimed at identification of the factors that control miR-19b, and analysis of the *in vivo* functions of miR-19b, will be required in order to clarify the significance of miR-19b regulation of oncogenic signaling pathways in cancer development.

## Methods

### Cell culture

293T and A2058 cells were obtained from the Japanese Collection of Research Bioresources Cell Bank (JRCB). CRL1579, SKMEL28 and G361 cells were obtained from the Cell Resource Center for Biomedical Research, Institute of Development, Aging and Cancer, Tohoku University., Japan. These cells were cultured in Dulbecco's modified Eagle's medium (DMEM; Sigma, St. Louis, MO, USA) supplemented with 10% fetal bovine serum (FBS; HyClone, Logan, UT, USA). HNEMs (Invitrogen, Gibco Cell Culture, Portland, OR, USA) were cultured in Medium 254 (Invitrogen, Gibco Cell Culture) supplemented with Human Melanocyte Growth Supplement (HMGS; Invitrogen, Gibco Cell Culture). All cells were cultured at 37°C in a humidified incubator with 5% CO_2_.

### Plasmid construction

#### pFLAG-control or pFLAG-PITX1

pFLAG-PITX1 was constructed by amplification of *PITX1* cDNA without the 3′UTR region from genomic DNA by PCR using KOD plus DNA polymerase (TOYOBO, Tokyo, Japan) and the following primer sequences: forward primer: 5′-GGAAGATCTTATGGACGCCTTCAAGGGGGGCATG, reverse primer: 5′-CGGGGTACCTCAGCTGTTGTACTGGCACGCGTTG, and was inserted into the BglII/Acc65I digested FLAG-tagged vector pCMV-FLAG4 (Sigma). The pCMV-FLAG4 vector was used as pFLAG-control.

### Western blotting

Western blotting was performed as described previously^4^. The membranes were blotted with a rabbit monoclonal antibody against the human PITX1 antigen (1:2,000; Abcam, Cambridge, MA, USA), a mouse monoclonal antibody against the FLAG antigen (1:2,000; Sigma), or with a polyclonal antibody against β-tubulin (1:5,000; Thermo Scientific, Tokyo, Japan) and the appropriate standard peroxidase-labeled anti-mouse IgG and anti-rabbit IgG secondary antibody according to the manufacturer's instructions (GE Healthcare, Piscataway, NJ, USA). Immunoreactive bands were visualized using the ECL detection system (Pierce, Rockford, IL, USA). PITX1 protein levels were quantified using Image J software.

### miRNA plasmid transfection

Cells were transfected with plasmid vectors using Lipofectamine 2000 (Invitrogen, Tokyo, Japan). For overexpression of miR-19a/b, 5 × 10^6^ 293T cells were seeded in each well of 6-well plates and were transfected 24 h after seeding with 0.5 µg of pCMV-miR19a (cloning pre-miR19a sequence in to pCMV-miR plasmid; GCAGUCCUCUGUUAGUUUUGCAUAGUUGCACUACAAGAAGAAUGUAGUUGUGCAAAUCUAUGCAAAACUGAUGGUGGCCUGC), pCMV-miR19b (cloning pre-miR19b sequence in to pCMV-miR plasmid; ACAUUGCUACUUACAAUUAGUUUUGCAGGUUUGCAUUUCAGCGUAUAUAUGUAUAUGUGGCUGUGCAAAUCCAUGCAAAACUGAUUGUGAUAAUGU) or pCMV-miR plasmid that also express the GFP gene that was used for fluorescence analysis of transfection efficiency (Origene, Rockville, MD, USA). The cells were harvested 48 h later. Stable cell lines were generated using a high amount of G418 (1 mg/mL).

### Oligonucleotide transfection

A2058 cells (1 × 10^5^ cells) were transfected with 90 pmol anti-miR-19b or negative control anti-miRNA oligonucleotides (Applied Biosystems, Tokyo, Japan, ID: MH10629) using Lipofectamine RNAiMAX (Invitrogen) according to the manufacturer's instructions.

### Cell proliferation assay

miR-19b and control stably transfected 293T cell clones (1 × 10^4^ cells/3ml/well), and anti-miR-19b and control oligonucleotide transfected A2058 cells (1 × 10^5^ cells/3ml/well), were seeded in 6-well culture plates. Cells were counted each day and the average cell number of three wells was quantified using a Coulter Counter Z2 (Beckman Coulter, Woerden, Netherlands)^48^.

### Luciferase assay

A segment of the human *PITX1* 3′UTR region containing the predicted binding site for miR-19b based on the TargetScan database (http://www.targetscan.org) was PCR-amplified from genomic DNA and inserted into the XbaI-digested pGL4.75 luciferase reporter vector (PITX1 3′UTR wt). The PCR primer sets designed to amplify full length of PITX1 3′UTR sequence. The PCR primers used were: forward primer: 5′- GCTCTAGAGCTTAGCACGGTCGGACTATGG, reverse primer: 5′- CTAGCTAGCTACCGCGGACCTCACACCTGC. To generate the PITX1 3′UTR mutated reporter (PITX1 3′UTR mut), a number of nucleotides in the PITX1 3′UTR that were complementary to the seed region nucleotides of miR-19b were mutated using PCR. The PCR primers used were: forward primer: 5′- TTACGTGTCCCGCTCTCCGGCCCGCGCCCCT, reverse primer: 5′- GTGGGCCGAGGGCCCTGGGGCCGCGCCCCT (the underline indicates mutated nucleotides). 293T cells were plated at a density of 1 × 10^6^ cells in 12-well plates 24 h before transfection. Reporter plasmids (0.05 µg) and miR expression plasmids (1.0 µg) were transfected using the Lipofectamine 2000 reagent (Invitrogen) according to the manufacturer's protocol. The cells were lysed 48 h after transfection and their luciferase activity was assayed using the Picagene dual SeaPansy luminescence kit (Toyo Ink, Tokyo, Japan) according to standard protocols. All experiments were performed independently three times. Luciferase activity was normalized to total protein concentration.

### qRT-PCR

RNA isolation and reverse transcriptase (RT)-PCR was performed as described previously^4^. miR-19b and mmu-miR-19b expression, and *PITX1, hTERT, mTert* mRNA expression, were detected using qRT-PCR. miR-19b expression was detected using Taq Man miR assay kits (Applied Biosystems, Assay ID: 000396, Assay Name: hsa-miR-19b) according to the manufacturer's protocol. MiR expression levels were normalized using snRNA U6 as a reference. The mRNA expression of *PITX1* and *hTERT* was analyzed using the specific primers: *PITX1:* forward; 5′-GCTACCCCGACATGAGCA, reverse; 5′-GTTACGCTCGCGCTTACG), *hTERT:* forward; 5′- GCCTTCAAGAGCCACGTC, reverse; 5′-CCACGAACTGTCGCATGT). The mRNA expression of *mTert* and mmu-miR-19b was analyzed using the specific primers: mmu-miR-19b*:* forward; 5′- TTGCAGATTTGCAGTTCAGCGT, reverse; 5′- TCCCACAATCAGTTTTGCATGG, *mTert:* forward; 5′- AGAGCTTTGGGCAGAAGGA, reverse; 5′- GAGCATGCTGAAGAGAGTCTTG). cDNA was amplified using an Applied Biosystems StepOne thermal cycler system and a SYBR green PCR kit (Applied Biosystems, Foster City, CA, USA). mRNA level was normalized to human *GAPDH* mRNA (PCR primers: forward; 5′-AGCCACATCGCTCAGACAC, reverse; 5′-GCCCAATACGACCAAATCC) for human genes and *mouse Gapdh* mRNA (PCR primers: forward; 5′-TCATTGTCATACCAGGAAATGAGC, reverse; 5′-GTCTCCTGCGACTTCAACAG) for mouse genes.

### Assay of telomerase activity

Telomerase activity was assayed by the stretch PCR method using the TeloChaser telomerase assay kit (TOYOBO, Osaka, Japan) as previously described methods^4^. To all the cells, 2.5 × 10^4^ cells were used for analysis. The products were electrophoresed on a 12.5% nondenaturing polyacrylamide gel and visualized by staining with SYBR Green I Nucleic Acid Gel Stain (Cambrex Bio Science, Rockland, ME, USA). Telomerase activity was quantified using Image J software.

### Tissue samples

Fourteen biopsy samples of human melanoma were obtained from the Tottori University Hospital. All the materials were obtained with written informed consent, and the procedures were approved by the institutional review board of Tottori University (Permission No.1558). All experiments were performed in accordance with guidelines of the Ethical Committee of Tottori University. Total RNA was extracted from the tissue samples using the Qiagen RNeasy plus kit (Qiagen, Tokyo, Japan), according to the supplier's instructions. Immunohistochemical analysis of PITX1 expression in these samples was performed as described previously^10^.

### Mouse tissue RNA isolation

C57BL/6 mice were obtained from Crea, Japan. Total RNA was isolated by Trizo Dnase-I treated and cDNA synthesised using MMLV-RT and random primers according to the manufacturer's protocols (Invitrogen). All animal experiments were approved by the Institutional Animal Care and Use Committee of Tottori University.

### Statistics

Data from more than two separate experiments are presented as means ± S.D. Significance was established at *P*-values less than 0.05 using an unpaired two-tailed Student's *t* test.

## Author Contributions

K.H. and T.O. designed the experiments and analyzed the data. T.O., N.S., Y.N., and M. Osaki performed the experiments and contributed to discussion. H.K. and T.O. wrote the manuscript. F.O. and M. Oshimura contributed to data analysis and discussion. H.K. conceived and managed the project. All authors revised and edited the manuscript.

## Supplementary Material

Supplementary Information Supplementary information
