# Supplementary material for: miR-19b regulates hTERT mRNA expression through targeting PITX1 mRNA in melanoma cells
Source: Sci Rep. 2015 Feb 3;5:8201. doi: 10.1038/srep08201 (PMC4314654; doi:10.1038/srep08201)
Supplement: Supplementary Information [file srep08201-s1.pdf]

Supplementary information

miR-19b regulates *hTERT* mRNA expression through targeting *PITX1* mRNA in melanoma cells

Takahito Ohira[1], Sunamura Naohiro[1], Yuji Nakayama[2], Mitsuhiko Osaki[3,4], Futoshi Okada[3,4], Mitsuo Oshimura[4], Hiroyuki Kugoh[1,4*]

[1]Department of Biomedical Science, Institute of Regenerative Medicine and Biofunction, Graduate School of Medical Science, Tottori University, Yonago, Tottori 683-8503, Japan

[2]Division of Functional Genomics, Research Center for Bioscience and Technology, Tottori University, Yonago, Tottori 683-8503, Japan

[3]Division of Pathological Biochemistry, School of Life Science, Faculty of Medicine, Tottori University, Yonago, Tottori 683-8503, Japan

[4]Chromosome Engineering Research Center, Tottori University, Yonago, Tottori 683-8503, Japan

[*]Correspondence should be addressed to H.K. (kugoh@med.tottori-u.ac.jp).
Department of Biomedical Science, Institute of Regenerative Medicine and Biofunction, Graduate School of Medical Science, Tottori University, 86 Nishi-Cho, Yonago, Tottori 683-8503, Japan, Tel: +81-859-38-6208. Fax: +81-859-38-6210.

Supplementary Figure S1.

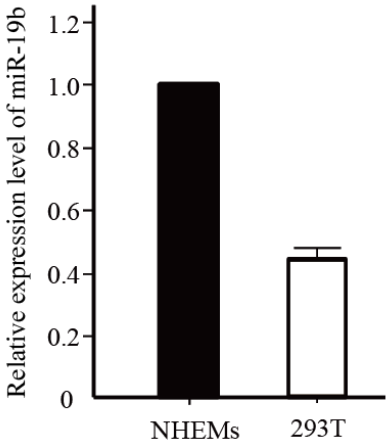

Supplementary Figure S1. Low expression of miR-19b in 293T cells.

qRT-PCR analysis of relative miR-19b expression levels in 293T cells and NHEMs. The expression level in NHEMs was arbitrarily assigned as 1. 293T cells displayed low expression of miR-19b. Data were normalized to U6 control. The bars correspond to means ± S.D. of three independent experiments.

Supplementary Figure S2.

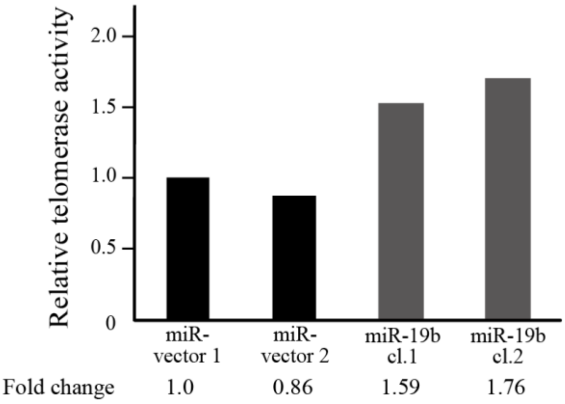

Supplementary Figure S2. Telomerase activity in stably express miR-19b clones.

Telomerase activity was measured using TeloChaser kit. Relative telomerase activity, which was quantitative by using Image J Imaging Software, calculated from the intensity of telomerase product bands.

Supplementary Figure S3.

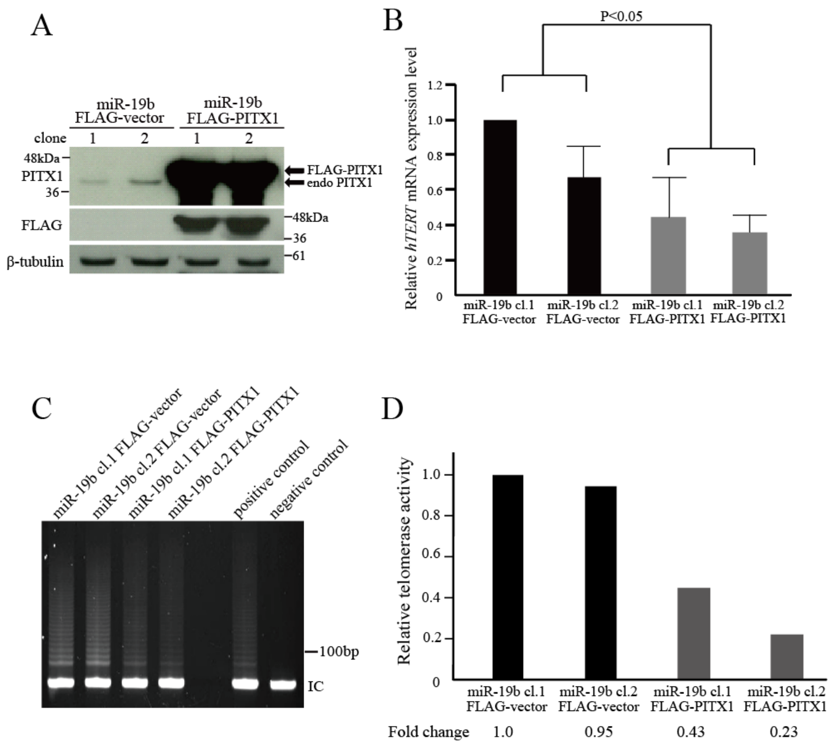

Supplementary Figure S3. Expression of exogenous *PITX1* suppressed *hTERT* expression in cells stably expressing miR-19b.

(A) Western blotting of endogenous and exogenous PITX1 in cell lines stably transfected with miR-19b and co-transfected with the FLAG-vector or with FLAG-PITX1. Endogenous and exogenous PITX1 were detected with anti-PITX1 and anti-FLAG antibodies respectively. (B) qRT-PCR analysis of *hTERT* mRNA expression level in the cells described in (A). *hTERT* mRNA expression level was decreased in the cells transfected with exogenous FLAG-PITX1 compared to that in FLAG-vector transfected cells. *hTERT* mRNA expression (*hTERT/GAPDH*) was calculated as the fold change relative to the control cl.1 cells, which were assigned a value of 1. The bars correspond to means ± S.D. of three independent experiments (*P<0.05). (C) Telomerase activity in in cell lines stably transfected with miR-19b and co-transfected with the FLAG-vector or with FLAG-PITX1. IC: internal control for PCR and loading. Telomerase activity of $2.5X10^4$ HeLa cells extract served as a positive control. Negative control was heat inactivated HeLa cell extract. (D) Relative telomerase activity, which was quantitative by using Image J Imaging Software, calculated from the intensity of telomerase product bands.

Supplementary Figure S4.

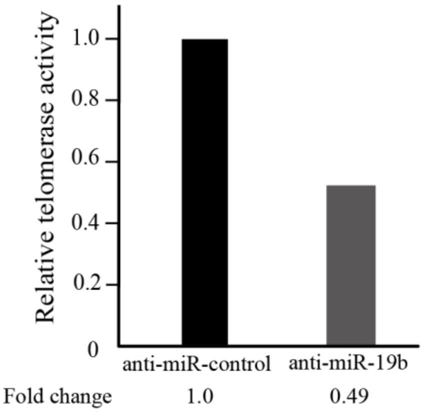

Supplementary Figure S4. Knockdown of miR-19b leads to inhibited telomerase activity. Telomerase activity in anti-miR control or anti-miR-19b oligonucleotide transfected A2058 cells. Relative telomerase activity, which was quantitative by using Image J Imaging Software, calculated from the intensity of telomerase product bands.

Supplementary Figure S5.

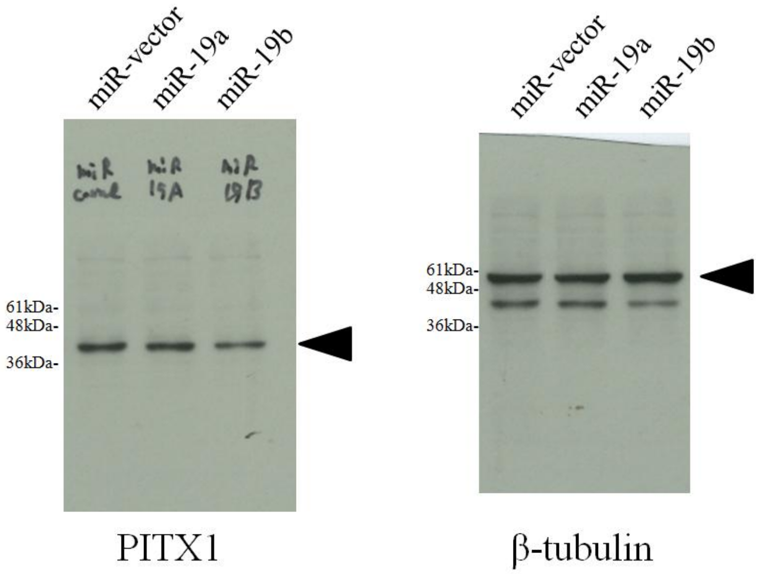

Supplementary Figure S5. Whole western blots from Figure 1D in the manuscript.

The left panel is the whole gel image for western blot using PITX1 antibody in Figure 1D. The right panel is the whole gel image for western blot using β-tubulin antibody in Figure 1D.

Supplementary Figure S6.

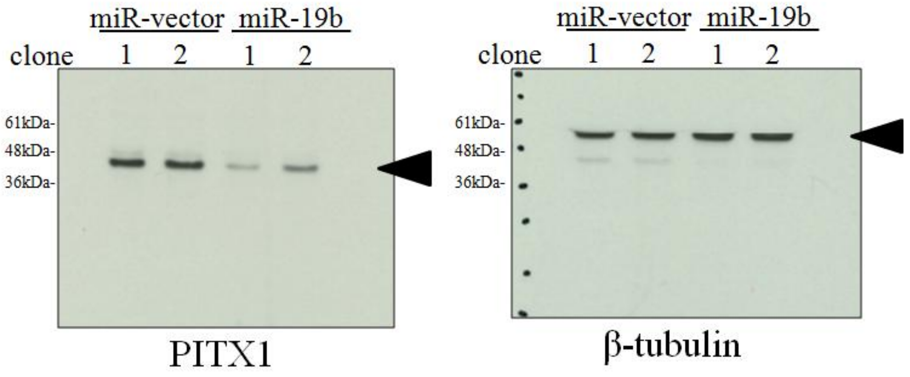

Supplementary Figure S6. Whole western blots from Figure 2C in the manuscript.

The left panel is the whole gel image for western blot using PITX1 antibody in Figure 2C. The right panel is the whole gel image for western blot using β-tubulin antibody in Figure 2C.

Supplementary Figure S7.

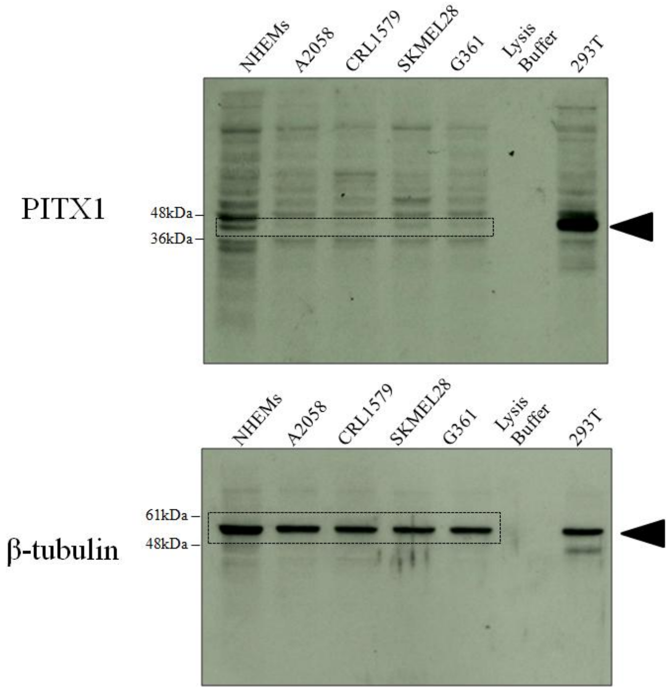

Supplementary Figure S7. Whole western blots from Figure 4A in the manuscript.

The top panel is the whole gel image for western blot using PITX1 antibody in Figure 4A. The bottom panel is the whole gel image for western blot using β-tubulin antibody in Figure 4A. 293T cell lysate was used as positive control.

Supplementary Figure S8.

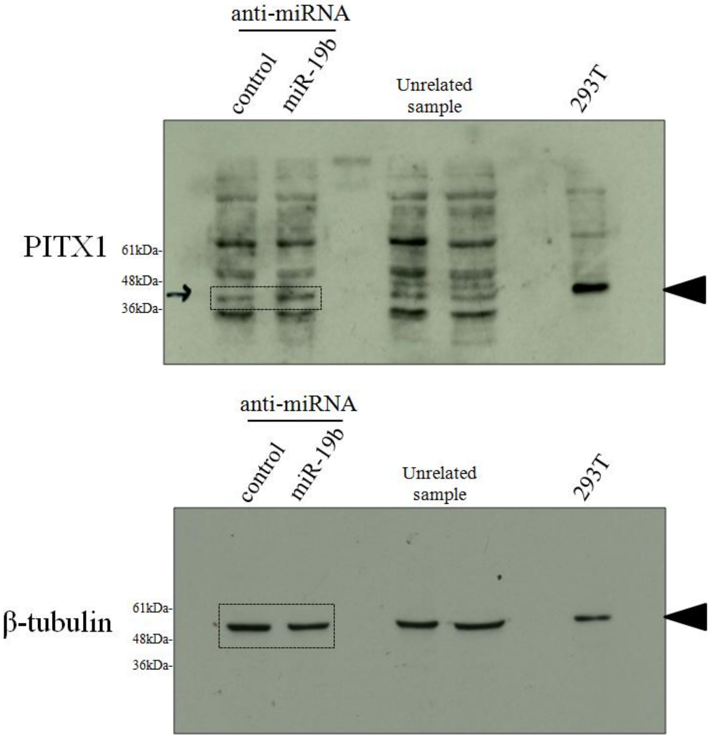

Supplementary Figure S8. Whole western blots from each Figure 4B in the manuscript. The top panel is the whole gel image for western blot using PITX1 antibody in Figure 4B. The bottom panel is the whole gel image for western blot using β-tubulin antibody in Figure 4B. 293T cell lysate was used as positive control.

Supplementary Figure S9.

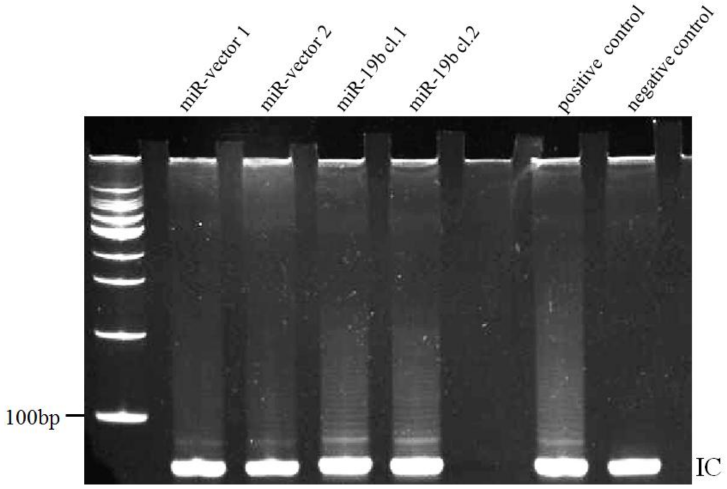

Supplementary Figure S9. Whole gel image from Figure 2E in the manuscript.

Supplementary Figure S10.

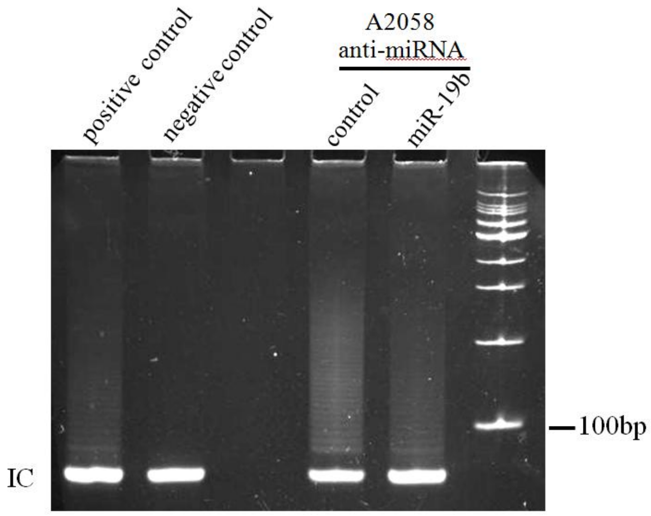

Supplementary Figure S10. Whole gel image from Figure 4E in the manuscript.
